# Genetics of Type 2 Diabetes: Past, Present, and Future

**DOI:** 10.3390/nu14153201

**Published:** 2022-08-04

**Authors:** Markku Laakso, Lilian Fernandes Silva

**Affiliations:** 1 Institute of Clinical Medicine, Internal Medicine, University of Eastern Finland, 70210 Kuopio, Finland; 2 Department of Medicine, Kuopio University Hospital, 70210 Kuopio, Finland

**Keywords:** type 2 diabetes, genetics, genome-wide association studies, precision medicine

## Abstract

Diabetes has reached epidemic proportions worldwide. Currently, approximately 537 million adults (20–79 years) have diabetes, and the total number of people with diabetes is continuously increasing. Diabetes includes several subtypes. About 80% of all cases of diabetes are type 2 diabetes (T2D). T2D is a polygenic disease with an inheritance ranging from 30 to 70%. Genetic and environment/lifestyle factors, especially obesity and sedentary lifestyle, increase the risk of T2D. In this review, we discuss how studies on the genetics of diabetes started, how they expanded when genome-wide association studies and exome and whole-genome sequencing became available, and the current challenges in genetic studies of diabetes. T2D is heterogeneous with respect to clinical presentation, disease course, and response to treatment, and has several subgroups which differ in pathophysiology and risk of micro- and macrovascular complications. Currently, genetic studies of T2D focus on these subgroups to find the best diagnoses and treatments for these patients according to the principles of *precision medicine*.

## 1. Introduction

Type 2 diabetes (T2D) has reached epidemic proportions worldwide. Currently, approximately 537 million adults (20–79 years) have diabetes, and the total number of people with diabetes is projected to increase to 643 million by 2030 and 783 million by 2045, according to the statistics of the International Diabetes Federation [1]. Diabetes is diagnosed by elevated fasting glucose, elevated 2 h glucose in an oral glucose tolerance test, or elevated hemoglobin A1c levels [2]. T2D reduces life expectancy and quality of life and increases the risk of macro- and microvascular complications [3].

A genetic component to T2D is important given the inheritance observed in families, the high prevalence for this disease in certain ethnic groups, and the difference in concordance rates between monozygotic and dizygotic twins [4]. The heritability of T2D has been reported to range from 30 to 70% [5]. Both insulin secretion and insulin action are impaired in T2D. Their relative importance has been debated, but it is now recognized based on genetic studies that β-cell dysfunction is the key factor in the development of this disease [6]. Genetic and environmental/lifestyle factors, especially obesity and sedentary lifestyle, increase the risk of T2D [3]. Several trials have reported that it is possible to delay or prevent T2D by healthy diet and physical activity [7].

The aim of this review is to discuss how studies on the genetics of diabetes started, how they expanded when genome-wide association studies (GWAS) and exome and whole-genome sequencing became available, and what the challenges for T2D are currently and may be in the near future. T2D is a heterogeneous disease but patients with T2D are currently treated as a homogeneous entity, although the current guidelines emphasize a personalized approach for diabetes treatment [8]. Recent studies have revealed new evidence that the identification of the subgroups of T2D may allow new tailored therapies for patients belonging to different subgroups of T2D in the near future.

## 2. Approaches in Studies of the Genetics of Diabetes

### 2.1. Development of Technologies for Genetic Studies

**Figure 1:**
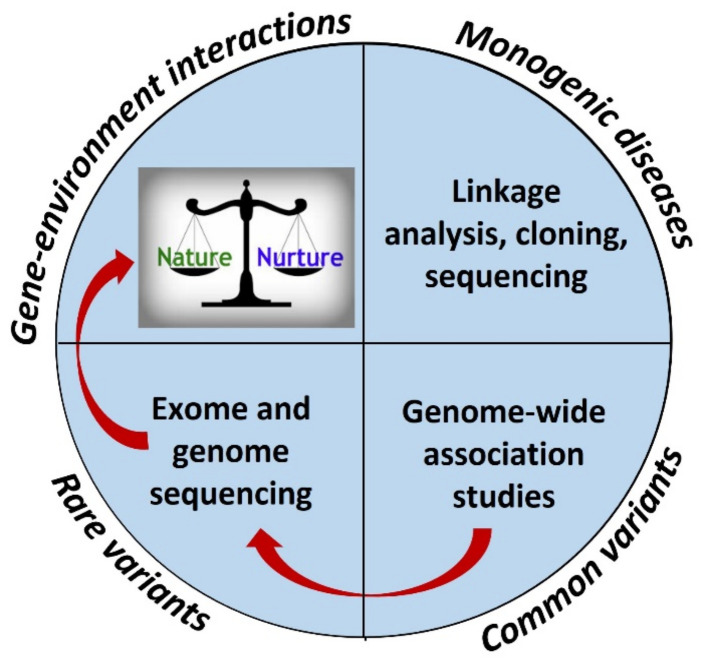
Phases in the studies of genetics of diabetes. Identification of monogenic diseases became possible after the development of cloning and sequencing. Genome-wide association studies made it possible to investigate the genetics of polygenic diseases, and exome and genome sequencing made it possible to identify rare variants. Gene–environment interaction studies involve both genetic and environmental effects.

Advances cloning, sequencing, genotyping, and analytical tools during the last 30 years made the studies of the genetics of different diseases possible. The Human Genome Project was instrumental for the development of genetic studies. This project constructed genetic and physical maps of the human genome, determined the sequence of human DNA, and identified the complete set of human genes [9]. In 1994, the human genetic linkage map fulfilled the first of the major goals when >100 laboratories published a comprehensive human linkage map [9]. That was the starting point for microsatellite-based genetic markers, and the development of statistical methods to analyze the data [10]. The next steps, microarray-based detection of structural variation and exome- and genome-wide sequencing methods by using new technology (Figure 1), were crucial for the development of genetic studies [11].

### 2.2. Candidate Gene Studies and Linkage Analyses

In the 1960s, it was believed that diabetes was a polygenic disorder. In 1975, it was discovered that young individuals with diabetes have autosomal dominant inheritance, and in the 1990s the first MODY (maturity-onset diabetes of the young) genes were identified [12,13,14]. However, studies of T2D were not successful by applying linkage-based approaches using multigenerational pedigrees and/or large numbers of affected sib-pairs. The next step to identify genetic variants for T2D was to use a candidate gene approach, most often these were case–control association studies [15]. These studies were usually small in size and very often reported conflicting results.

**Figure 2:**
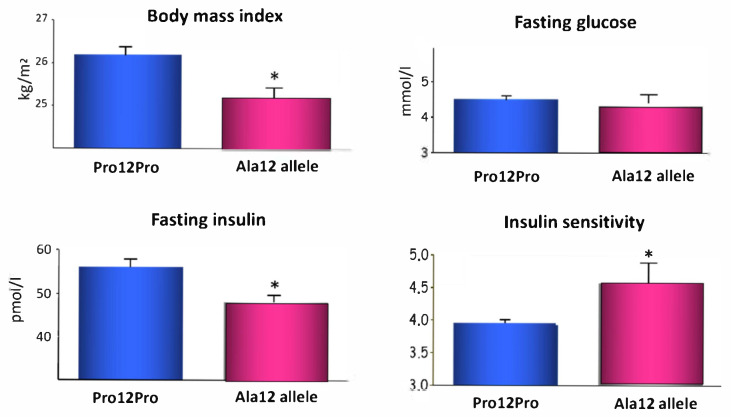
Body mass index and fasting insulin were significantly decreased and insulin sensitivity was increased (* *p* < 0.05) in the carriers of the Ala12 allele of *PPARG2*.

The first success in the application of a candidate gene approach in T2D studies was our study where we investigated the *PPARG* gene in 1998 [16]. PPARγ1 and PPARγ2 have effects on energy balance and body mass index (BMI), and we hypothesized that PPARγ may constitute a predisposing factor for obesity and insulin resistance. Further evidence for the significance of the *PPARG* gene came from the drug treatment of patients with T2D because troglitazone, a PPARγ agonist, lowered blood glucose concentrations [17]. We found that Pro12Ala substitution in *PPARγ2* was significantly associated with lower BMI and insulin concentration and improved insulin sensitivity among middle-aged Finns (Figure 2). We also found that the Pro allele of *PPARγ2* was significantly associated with an increased risk of T2D among Japanese Americans [16]. In another study, we demonstrated that Pro12Ala knock-in mice on chow diet were leaner and had improved insulin sensitivity and plasma lipid profiles [18]. Our results suggest that Pro12Ala of *PPARG* is an important modulator of metabolic control.

Altshuler et al. [19] performed a meta-analysis of 16 separate studies including over 3000 participants, and showed that the Pro allele of *PPARG* was significantly associated with the risk of T2D. This study is unique because it demonstrated for the first time that meta-analysis is needed to increase the sample size to obtain statistically significant and reliable results. It also showed that linkage analysis is not suitable for discovering the impact of common-risk alleles. Therefore, the genetic dissection of polygenic diseases needs association studies performed on large population samples.

In 1998, Hani et al. published the first genetic variant identified by the candidate gene approach associated with impaired insulin secretion [20]. They identified an amino acid substitution of Glu23Lys in the *KCNJ11* gene in three Caucasian cohorts and showed that this variant was associated with the risk of T2D. The study by Gloyn et al., published in 2003, included 854 patients with T2D and 1182 controls and reported an 18% increase in the risk of T2D in the carriers of Glu23Lys of *KCNJ11* [21]. Barroso et al. investigated 71 candidate genes for T2D in 2134 Caucasians and found 15 genetic variants potentially important for the risk of T2D but were not able to confirm these findings in meta-analysis of several cohorts [15]. However, this study made an important observation that genetic variants were more often associated with decreased insulin secretion than with insulin sensitivity [15].

In general, linkage analysis alone was not successful to identify genes for the risk of T2D. Grant et al. identified the *TCF7L2* locus in 2006 as a risk gene for T2D using the combination of linkage analysis and genotyping microsatellite markers across the chromosome 10q region in a study that included 1185 individuals with T2D and 931 controls [22]. This finding was confirmed one year later in a French case–control cohort for the T allele of a single-nucleotide variant (rs7903146) of *TCF7L2* [23]. In a meta-analysis comprising 28 studies, this intronic variant of *TCF7L2* increased the risk of T2D by 41%, which is the most statistically significant single variant among all risk variants for T2D [24].

### 2.3. Genome-Wide Common Variant Association Studies

Genome-wide association studies (GWAS) have been successful in identifying common variants that increase the risk of T2D. The first studies, published in 2007, included thousands of participants and identified 10 genome loci exploiting single-nucleotide polymorphism microarrays [23,25,26,27,28]. Importantly, many of these studies identified the same variants. These studies also showed that all common variants identified by GWAS increased the risk of T2D < 40%, and most of them by only < 15%.

The next step in common variant GWAS studies was to share the data across the studies to increase the statistical power. This made it possible to identify more variants with small effects on the risk of T2D [29]. The Diabetes Genetics Replication and Meta-analysis Consortium (DIAGRAM) increased sample size to above 10,000 for case–control studies [30] and the second DIAGRAM up to 45,000 including participants of European ancestry [31]. Collaboration of large consortia resulted in the development of a custom genotyping array that made it possible to increase the sample size up to 150,000 in GWAS studies [32].

GWAS studies have provided important information about the genetic architecture of T2D. Voight et al. [33] and Ingelsson et al. [34] reported that the variants associated with the risk of T2D were more often associated with decreased insulin secretion than insulin sensitivity. Scott et al. [35] reported three pathological groups for T2D: impaired insulin secretion/insulin processing, insulin resistance, and dyslipidemia. Lotta et al. [36] generated a genetic risk score for a lipodystrophy-like subset of T2D. These studies were instrumental to understand the heterogeneity of T2D.

A study of Mahajan et al. [37] included 74,124 cases of T2D and 824,006 controls of European ancestry and identified 403 distinct association signals. This study also highlighted potential for clinical translation given the fact that genome-wide chip heritability explained 18% of T2D risk. These authors also developed a polygenic risk score (PRS) and applied it to the general UK population and estimated that the PRS predicts a lifetime T2D risk of 59.7% in individuals < 55 years of age.

Diabetes Meta-Analysis of Trans-Ethnic (DIAMANTE) association studies included not only Europeans but also non-European populations. This T2D study, which included 228,499 T2D cases and 1,178,783 controls from five ancestral groups, is the largest GWAS study published so far about the variants associated with T2D [38]. The authors reported 568 associations and 318 novel risk loci for T2D. They performed pathway and functional enrichment analysis and found that the most significant gene set involved the AKT2 subnetwork, a gene associated with the risk of T2D. The authors also reported novel findings on the complications of T2D. Their PRS was strongly associated with an increased risk of T2D-related retinopathy [38].

GWAS studies have been highly successful and have so far reported > 700 novel T2D risk loci. These studies demonstrate that increased sample size and inclusion of participants from diverse ancestral backgrounds substantially increase statistical power to identify new association signals. Consequently, the effect size of novel risk variants for T2D has decreased, indicating that these variants can be statistically significant but their contribution to the understanding of the pathophysiology of T2D is limited [39].

Diabetes is defined by elevated concentrations of glycated hemoglobin A1c (HbA1c), fasting glucose, or 2 h glucose. HbA1c measures average glycemia over the period of the last 2–3 months, whereas fasting and 2 h glucose levels change daily. Multiple GWAS studies have been published on genetic variants associated with HbA1c since 2008 [40,41]. The most recent Meta-Analyses of Glucose and Insulin-related Traits Consortium (MAGIC) included >280,000 individuals of diverse ancestry without diabetes, and reported associations of variants with glucose, insulin, and HbA1c [42]. In this study, 218 HbA1c-associated variants were reported. The authors generated a PRS including all HbA1c-associated signals and showed that it was strongly associated with an increased risk of T2D.

Several studies have reported significant associations of genetic variants with fasting glucose [42,43,44,45,46,47], fasting insulin [42,43,44,45], fasting proinsulin [48], and insulin resistance [43]. The largest study on glycemic traits published by Chen and collaborators [42] included 281,416 individuals without diabetes (70% European ancestry, 30% non-European ancestry). They identified a total of 242 loci (99 novel) for HbA1c, fasting 2 h glucose, and fasting insulin. Walford et al. reported that *BCL2* and *FAM19A2* are novel insulin sensitivity loci [49].

### 2.4. Genome-Wide Rare Variants Association Studies

In 2013, we published the first study investigating the significance of low-frequency variants (<5%) to the risk of T2D or T2D-related traits [50]. Our study included 8229 Finns and used the Illumina exome array. We reported two low-frequency variants associated with fasting proinsulin concentrations (*SGSM2*, *MADD*), and three novel variants (*TBC1D30*, *KNK1*, *PAM*) associated with proinsulin or insulinogenic index. Our study provided proof of the principle that exome genotyping array identifies low-frequency functional variants that contribute to complex traits. In 2014, Steinthorsdotter et al. [51] published a genome sequencing study in Icelanders and found three more T2D-associated low-frequency variants (*CCND2*, *PAM*, *PDX1*). During the following years, rare variants associated with T2D or T2D-related traits in *MTNR1B*, *HNF1*, and *G6PC2* [52,53] were published.

The first loss-of-function variant protective of T2D was published by Flannick et al. in 2014 [54]. They sequenced or genotyped ~150,000 participants from five ancestry groups and identified several rare loss-of-function variants in *SLC30A8* encoding an islet zinc transporter. The Trp325Arg variant of this gene was protective against T2D (65%).

Interestingly, a partial loss-of-function rare *AKT2* variant Pro50Thr [55] was almost entirely specific to Finns (frequency 1.1%). This gene regulates insulin signaling and insulin sensitivity and increases the risk of T2D. We measured the whole-body and tissue-specific insulin sensitivity with positron emission tomography in 20 carriers and 25 matched controls [56]. We found a 39% decrease in whole-body glucose uptake and a 56% increase in the rate of liver glucose production. Glucose uptake was significantly reduced in multiple tissues, including liver, skeletal muscle, brown adipose tissue, and bone marrow. We also found that glucose uptake was increased significantly in all seven tested brain regions. Our study demonstrates that the Pro50Thr variant of *AKT2* has effects on insulin-mediated glucose uptake in multiple insulin-sensitive tissues. Our study shows that rare variants can provide significant information about gene function and reveal novel information about glucose metabolism.

### 2.5. Polygenic Risk Scores for Type 2 Diabetes

GWAS studies have made it possible to generate PRSs which estimate an individual’s lifetime genetic risk for different diseases [57]. Earlier onset of the disease may be caused by increased genetic risk. Therefore, PRSs have the potential to improve the likelihood of preventing chronic diseases [58]. Several studies on coronary artery disease have reported that disease-prediction algorithms perform better when PRSs are added to models having clinical risk factors [59]. However, the contribution of PRS is substantially less in prediction models for T2D. The area under the receiver operating characteristics (ROC) curve (AUC) is a measure of a prediction accuracy of a PRS [60].

The first studies using PRSs to increase the prediction of the risk of T2D beyond clinical risk factors included 16–18 genetic variants which were significantly associated with T2D [61,62,63]. All these studies showed that PRS increased the risk prediction of T2D only marginally. Vassy et al. [64] included 62 genetic variants in their PRS, which improved T2D prediction compared with previous studies. Our study on 8749 Finnish men included a PRS with 76 genetic variants [65]. When we added this PRS into a prediction model consisting of clinical and laboratory risk factors for T2D (age, BMI, smoking status, physical activity, HDL cholesterol, triacylglycerol, and systolic blood pressure), we found that our PRS improved the prediction of T2D only slightly (AUC increased from 0.711 to 0.719).

Previous studies have been too small to realize the full potential of PRSs in T2D risk prediction since they may miss a large proportion of cases (>50%) by targeting only high-risk individuals. A 10-fold increase in sample size (about 220,000) in a GWAS study by Chatterjee et al. [66] substantially increased the performance of PRSs. Thus, PRSs based on genetics can help in the estimation of disease risk and in planning of clinical applications. Recommendations have been published to improve reporting standards for PRSs in risk prediction studies [67].

## 3. Precise Type 2 Diabetes Medicine

### 3.1. Genetics

Precision medicine requires that prevention and treatment strategies account for individual variability. Current guidelines advocate a personalized approach for diabetes treatment [68]. Application of this concept has been improved by recent developments in genetics of the human genome sequence, powerful methods for characterizing patients (proteomics, metabolomics, cellular assays), and computational tools to analyze large databases [69].

**Figure 3:**
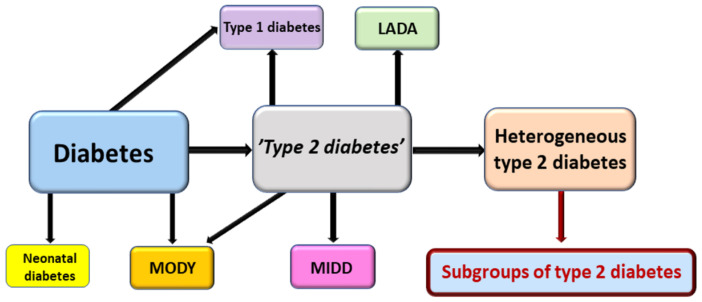
Identification of the subgroups of type 2 diabetes. Diabetes has several subtypes which need to be excluded when analyzing the subgroups of type 2 diabetes. Neonatal diabetes, maturity onset diabetes of the young (MODY), and type 1 diabetes are diagnosed at young age; latent autoimmune diabetes in adults (LADA) and mitochondrial diabetes and deafness (MIDD) in middle or elderly age. When all subtypes of diabetes have been excluded we have heterogeneous type 2 diabetes, and the subgroups can be identified.

Diabetes is defined by elevated glucose levels, either in the fasting state or postprandially. The most common type of diabetes is T2D, which accounts for about 80% of all cases. Type 1 diabetes occurs in about 10% of cases, and latent autoimmune diabetes of the adult (LADA) occurs in about 5% of cases [70]. MODY and other monogenic forms of diabetes and secondary diabetes cover the rest of diabetes cases. T2D does not have an accurate definition, and therefore it is a diagnosis of exclusion. Quite often studies on T2D include patients with misdiagnosed forms of diabetes, especially LADA and type 1 diabetes. Therefore, it is important to identify and exclude other diabetes subtypes when investigating the subgroups of T2D (Figure 3).

T2D itself is a heterogeneous disease with respect to clinical presentation, disease course, and response to treatment. T2D has several subgroups which differ in pathophysiology and risk of micro- and macrovascular complications. The first effort to identify T2D subgroups was published by Li et al. [71]. Their aim was to identify T2D subgroups by topological analysis of patient similarity based on electronic medical records and genotyping. They reported three subgroups of T2D for diabetic micro- and macrovascular complications. Subtype 1 was characterized by retinopathy and diabetic nephropathy; subtype 2 by cardiovascular diseases and cancer; and subtype 3 by neurological diseases, cardiovascular diseases, and allergies [71]. These authors also performed an association analysis of the T2D subgroups to find subtype-specific genetic markers and reported several genetic variants for subgroups 1, 2, and 3. The limitation of this study is that they did not focus on the pathophysiology of T2D or genetic variants associated with the risk of this disease.

Ahlqvist et al. [72] proposed a new classification of T2D based on cluster analysis of the following six clinical traits and laboratory tests: age, BMI, HbA1c, GAD antibodies, HOMA2-B (a measure of insulin secretion), and HOMA-IR (a measure of insulin resistance). The first cluster was severe autoimmune diabetes (SAID) defined by positive GAD antibodies, including type 1 diabetes and LADA (6–8% of adult individuals), characterized by decreased insulin secretion, low/normal BMI, and poor metabolic control. The second cluster, severe insulin-deficient diabetes (SIDD), had similar characteristics as SAID with respect to impaired insulin secretion and poor glucose control but did not have positive GAD antibodies (18–20%). The third cluster, severe insulin-resistant diabetes (SIRD), was characterized by high insulin resistance as measured by HOMA2-IR, HOMA2-B, high BMI, and low HbA1c. The fourth cluster, mild obesity-related diabetes (MOD), was characterized by high BMI at a relatively young age (‘heathy obesity’) but not insulin resistance (20–25%), and the fifth cluster, mild age-related diabetes (MARD), was characterized by the latest onset of diabetes and low BMI.

A recent study by Aly et al. [73] investigated the significance of genetic variants in the classification of T2D into subgroups originally identified by Ahlqvist et al. [72]. The five subgroups of T2D differed with respect to diabetes-related traits and family history of diabetes. SIRD was associated with PRSs for fasting insulin and diabetes. Three subgroups of T2D, SAID, SIDD, and SIRD, had partially distinct pathophysiology. MOD-specific *LRMDA* locus was found, and therefore it can be concluded that subclassification of T2D may improve the power to detect diabetes loci.

Intermediate phenotypes (body mass index, fasting insulin, lipid levels, etc.) have been recently used to account for the observed clinical heterogeneity in the identification of subgroups of T2D [74]. These “partitioned genetic risk scores” have the potential to identify patients at high risk of T2D or rapid disease progression. They also help in stratifying subtypes of different diseases and bridging the gap toward precision medicine.

The study by Udler et al. [75] was based primarily on germline genetic variants. This study categorized 94 T2D genetic variants into subgroups representing disease mechanistic pathways and investigated whether these clusters of variants have important effects on 47 diabetes-related metabolic traits. The investigators found five robust clusters of T2D. The first two clusters were related to beta cell function in the pancreas. The three other clusters were related to insulin resistance and are mediated by obesity, fat-distribution (lipodystrophy) [76], and liver lipid metabolism. PRSs of top-weighted loci from the five clusters were associated with increased risk of coronary artery disease, stroke, and elevated systolic blood pressure. A recent study [77] reported that increased obesity and lipodystrophy cluster were significantly associated with hypertension and elevated blood pressure. The lipodystrophy and liver/lipid cluster included genetic variants of *GCKR, PNPLA3*, and *TM6SF2*, and were significantly associated with coronary artery disease. Additionally, the liver/lipid cluster was significantly associated with decreased renal function.

Wagner et al. [78] investigated intermediate hyperglycemia as an indication of elevated risk of developing T2D. Their study included participants from a cohort of individuals at high risk of T2D. The measurements included oral glucose tolerance tests, MRI-measurements of liver fat content and body fat distribution, and genetic risk. They could identify six clusters of sub-phenotypes, and in three of these clusters the participants had elevated glucose concentrations. However, in only two of these clusters were the participants at high risk of developing T2D. Interestingly, the participants belonging to a cluster having moderate risk of T2D had an increased risk of kidney disease and mortality. This study suggests that there is pathophysiological heterogeneity among individuals in the prediabetes stage.

A recent study by Wesolowska-Andersen et al. [79] included 726 participants of the DIRECT study. They applied a soft-clustering method (archetype) to characterize newly diagnosed patients with T2D and found four archetype profiles. One archetype was characterized by obesity, dyslipidemia, insulin resistance, and impaired β-cell glucose sensitivity, and these participants had the fastest disease progression. Similarly, another recent study [78] demonstrated clinical heterogeneity in the conversion to T2D. However, this study has limitations because it is small in size and the replication of the results is missing.

In summary, the first studies aiming to understand the heterogeneity of T2D have been published. It is not surprising that impaired insulin secretion and insulin resistance or their combination are important subgroups of T2D. Udler et al. [75] were the first to show that the liver/lipids subgroup of T2D is its own entity. This observation is supported by previous studies demonstrating that *TM6SF2* is associated with T2D [32] and non-alcoholic fatty liver disease (NAFLD) [80].

### 3.2. Phenotyping

The precision medicine approach requires a better understanding of both the genome and the phenome. Development of phenotype measurements is important for tailoring of individualized treatment to each patient. Phenotype characterization is especially important for polygenic diseases, including T2D, because both genetic factors and environmental/lifestyle factors determine the risk, whereas in monogenic diseases the risk is determined almost entirely by the causal genetic variants.

There are obvious gaps in our understanding of gene–environmental/lifestyle interactions related to the risk of T2D [81]. Recent studies have demonstrated that the classification of patients with T2D into subgroups needs a combination of both genetic variants and detailed phenotype. Decreased insulin secretion and insulin sensitivity [82] are the hallmarks of the conversion to T2D and, therefore, the most reliable indices for measuring insulin secretion and insulin sensitivity should be applied.

Several studies have been published about different laboratory measurements and other biomarkers as risk factors for T2D. These association studies do not, however, prove causality [83]. Mendelian Randomization (MR) studies could identify causal associations. In this method, common genetic variants are applied as instruments to estimate the causal effects of a risk factor on an outcome [84]. MR studies have confirmed that obesity [85] and the waist/hip ratio [86] are causal for the risk of T2D.

Metabolomics has been applied to studies on T2D in several population studies in recent years [87]. Potentially, new metabolites and pathways can characterize pathophysiological alterations in T2D [88,89]. Unfortunately, these studies have often been too small, and the number of metabolites determined in these studies has often been <200 compared to the thousands of metabolites available [83].

Therefore, the potential of the metabolomics approach has not been fully determined. Metabolomics combined with the MR approach could identify causal metabolites for T2D that could considerably improve prediction models. Similarly, proteomics provides valuable insights into how genetic and environmental/lifestyle factors are linked to clinical outcomes. Population-scale analyses of proteomics are currently largely missing, but they may reveal novel drug targets and biomarkers for metabolic diseases, including T2D [90].

## 4. Conclusions

During recent decades, our knowledge of the genetics of monogenic and polygenic forms of diabetes has experienced tremendous advancements. Consequently, in monogenic diabetes subtypes, MODY, and neonatal diabetes, the precision medicine approach of tailoring treatment to the individual characteristics of each patient has been successfully applied [91]. In contrast, in polygenic diabetes subtypes, and especially in T2D, the identification of the subgroups is challenging and currently, implications for patient care are largely missing. However, the PRSs predict the risk of T2D, and combined with relevant phenotypes, they are likely to show the way for improving the understanding of pathophysiology of the subgroups of T2D.

## Data Availability

Not applicable.
